# Wireless versus routine physiologic monitoring after cesarean delivery to reduce maternal morbidity and mortality in a resource-limited setting: protocol of type 2 hybrid effectiveness-implementation study

**DOI:** 10.1186/s12884-021-03550-w

**Published:** 2021-02-12

**Authors:** Adeline A. Boatin, Joseph Ngonzi, Blair J. Wylie, Henry M. Lugobe, Lisa M. Bebell, Godfrey Mugyenyi, Sudi Mohamed, Kenia Martinez, Nicholas Musinguzi, Christina Psaros, Joshua P. Metlay, Jessica E. Haberer

**Affiliations:** 1 Department of Obstetrics and Gynecology, Massachusetts General Hospital, 55 Fruit Street, Founders 5, Boston, MA USA; 2 Center for Global Health, Massachusetts General Hospital, Boston, MA USA; 3 Harvard Medical School, Boston, USA; 4 Program for Global Surgery and Social Change, Boston, USA; 5 Department of Obstetrics and Gynecology, Mbarara University of Science and Technology, Mbarara, Uganda; 6 Department of Obstetrics and Gynecology, Beth Israel Deaconess Medical Center, Boston, MA USA; 7 Department of Medicine, Massachusetts General Hospital, Boston, MA USA; 8 Global Health Collaborative, Mbarara University of Science and Technology, Mbarara, Uganda; 9 Department of Psychiatry, Massachusetts General Hospital, Boston, MA USA

**Keywords:** Wireless physiologic monitoring, Post-operative monitoring, Cesarean delivery, Hybrid effectiveness-implementation trial, Maternal mortality

## Abstract

**Background:**

Women in sub-Saharan Africa have the highest rates of morbidity and mortality during childbirth globally. Despite increases in facility-based childbirth, gaps in quality of care at facilities have limited reductions in maternal deaths. Infrequent physiologic monitoring of women around childbirth is a major gap in care that leads to delays in life-saving interventions for women experiencing complications.

**Methods:**

We will conduct a type-2 hybrid effectiveness-implementation study over 12 months to evaluate using a wireless physiologic monitoring system to detect and alert clinicians of abnormal vital signs in women for 24 h after undergoing emergency cesarean delivery at a tertiary care facility in Uganda. We will provide physiologic data (heart rate, respiratory rate, temperature and blood pressure) to clinicians via a smartphone-based application with alert notifications if monitored women develop predefined abnormalities in monitored physiologic signs. We will alternate two-week intervention and control time periods where women and clinicians use the wireless monitoring system during intervention periods and current standard of care (i.e., manual vital sign measurement when clinically indicated) during control periods. Our primary outcome for effectiveness is a composite of severe maternal outcomes per World Health Organization criteria (e.g. death, cardiac arrest, jaundice, shock, prolonged unconsciousness, paralysis, hysterectomy). Secondary outcomes include maternal mortality rate, and case fatality rates for postpartum hemorrhage, hypertensive disorders, and sepsis. We will use the RE-AIM implementation framework to measure implementation metrics of the wireless physiologic system including *Reach* (proportion of eligible women monitored, length of time women monitored), *Efficacy* (proportion of women with monitoring according to Uganda Ministry of Health guidelines, number of appropriate alerts sent), *Adoption* (proportion of clinicians utilizing physiologic data per shift, clinical actions in response to alerts), *Implementation* (fidelity to monitoring protocol), *Maintenance* (sustainability of implementation over time). We will also perform in-depth qualitative interviews with up to 30 women and 30 clinicians participating in the study.

**Discussion:**

This is the first hybrid-effectiveness study of wireless physiologic monitoring in an obstetric population. This study offers insights into use of wireless monitoring systems in low resource-settings, as well as normal and abnormal physiologic parameters among women delivering by cesarean.

**Trial registration:**

ClinicalTrials.gov, NCT04060667. Registered on 08/01/2019.

**Supplementary Information:**

The online version contains supplementary material available at 10.1186/s12884-021-03550-w.

## Background

Reducing maternal mortality continues to be a global reproductive health priority. Between 1990 and 2015, global maternal mortality dropped by 45%, from 532,000 deaths per year to an estimated 303,000 [1, 2]. However, childbirth remains a period of great risk for women and their babies in many resource-limited settings, with 295,000 maternal deaths and 6 million perinatal deaths (i.e., fetal and neonatal) occurring annually [3, 4]. Most maternal deaths occur around the time of delivery and approximately 45% within the first 24 h [5, 6]. As such, the promotion of facility-based childbirth has been a cornerstone of strategies to reduce maternal and neonatal deaths in low and middle-income countries [7, 8]. However, despite increases in the proportion of women delivering at facilities, reductions in mortality and morbidity have not been as rapid as expected. Increasing evidence for this gap points to poor quality of care received at facilities as a contributing factor [7]. The Lancet Commission for High Quality Health Systems reports that up to 55% of maternal deaths occur due to poor quality of care received, rather than non-utilization of care [9].

High quality facility-based intrapartum and postpartum care relies on physiologic monitoring as a strategy to identify potential obstetric complications and to guide management if complications occur [10]. The top three causes of maternal mortality, hemorrhage, hypertensive disease and sepsis, are commonly associated with abnormalities in physiologic signs [11]. Tachycardia and hypotension are clinical features of hemorrhage; hypertension and hypoxia are features of pre-eclampsia; and fever, tachycardia, hypotension and hypoxia are features of sepsis [12, 13]. Thus monitoring for and responding to these abnormalities is a key feature of preventing maternal morbidity and mortality. In emergency and medicine wards, abnormalities in physiologic signs have been shown to predict subsequent in-hospital mortality and morbidity [14–18]. In response to these findings, health care systems in resource-rich settings have introduced “Track and Trigger” protocols or “Early Warning Scores” to identify abnormalities in physiologic signs and activate an appropriate response [19]. Combining early warning of potential illness with appropriate medical response has reduced rates of cardiac arrest, readmissions to critical care and mortality [20–22]. Similar to early warning scores for emergency and medicine wards, national patient safety groups in the UK and United States advocate for the adoption of maternal early warning systems to improve recognition and prevention of serious obstetric morbidity and mortality through improved utilization of physiologic signs [23, 24]. These systems have been validated for the prediction of serious illness in obstetric populations [25], including a study in India where an adapted maternal early warning system was found to have a sensitivity of 86% and positive predictive value of 53% for obstetric morbidity [12].

However, physiologic monitoring is resource intensive, particularly for intrapartum and postpartum care. The World Health Organization calls for “close” monitoring in the immediate postpartum period [26]. Nursing standards by professional organizations in resource-rich settings operationalize “close” monitoring as physiologic monitoring every 15 min for the first 2 h postpartum, then every fours for the first 24 h [10, 27]. Traditional methods of physiologic monitoring typically require a nurse or nursing assistant to go to a patient’s bedside and obtain the different physiologic parameters. With this standard of care, sustaining the recommended high frequency of monitoring is a challenge even in resource-rich settings [28, 29]. In resource-limited settings, this is unachievable. In a study testing the functionality and acceptability of a wireless maternal vital sign monitor in Mbarara Hospital in southwestern Uganda, it was found that the physician to patient ratio ranged from 1:8 during the day to 1:14 during the night while the nursing/midwife to patient ratios are approximately 1:25 during the day and 1:50 at night [30]. In another study, examining physiologic monitoring during intrapartum care in Mbarara, Uganda, we found that less than 1% of women had high quality postpartum physiologic monitoring defined as the assessment of maternal blood pressure, heart rate and temperature within 4 h of delivery, and less than 4% at 24 h after delivery [31]. Our findings mirror those of one of the largest interventional studies for quality improvement around obstetric care in a resource-limited setting that tested the use of a checklist for improving quality; maternal blood pressure and temperature were checked in less than 38% of women in the intervention arm, and less than 3% of women in the control arm, at any point during their obstetric admission [32].

There are few pragmatic trials of strategies to improve the frequency and accuracy of physiologic monitoring in resource-limited settings. To date, these have relied on hand-held vital sign monitors that require bedside acquisition of physiologic parameters by staff [33]. In many resource-limited settings, where nurse to patient ratios may be as high as 1:50, such one-to-one monitoring is prohibitive. Advancements in wireless physiologic monitoring offer a novel strategy to improve the ability to obtain and react to abnormalities in physiologic signs. Wireless physiologic monitoring systems comprise a wearable wireless biosensor that acquires physiologic data, transmits data over a wireless network to cloud storage. Data can be transmitted from cloud storage to a central processing unit, web interface, or to other app-enabled devices (e.g. smart phone) for review by a clinician. In prior pilot work, we have shown that this type of technology is functional in both resource-rich and resource-limited settings and acceptable to both clinicians and pregnant women [34, 35].

We aim to expand on this pilot work and evaluate the clinical effectiveness and implementation of a wireless physiological monitoring system for immediate postpartum monitoring in a resource limited setting. To do so, we will conduct a type 2 hybrid effectiveness-implementation trial. Type 2 hybrid designs test clinical effectiveness and implementation simultaneously and with equal priority. Our primary aims are to 1) estimate the clinical effectiveness of wireless physiologic monitoring in the first 24 h after delivery in postpartum women at a regional referral and teaching hospital in a resource-limited setting and 2) evaluate measures of implementation of wireless physiologic monitoring in this setting.

## Methods

### Study design

This is a pragmatic type 2 hybrid effectiveness-implementation trial utilizing a quasi-experimental, interrupted time series with repeated on/off periods approach and carried out in a single facility.

### Study setting

The study will be carried out in the Department of Obstetrics and Gynecology (OB/GYN) at the Mbarara Regional Referral Hospital (MRRH), which is the primary referral hospital for southwestern Uganda and the teaching hospital for Mbarara University of Science and Technology. MRRH is a publicly funded 600-bed hospital. Hospital records indicate approximately 9000 deliveries per year, a average maternal mortality ratio of 389 per 100,000 live births, and a cesarean delivery rate of 37% between 2013 and 2018 (unpublished data). Approximately 21% of patients are referred to the institution from other health facilities. Inpatient services in this department include antenatal management of high-risk pregnancies, intrapartum care, postpartum care, and gynecologic surgery management. The unit is staffed by 24 nurse-midwives, 13 consultant OB/GYNs, and 30 post-graduate doctors training in OB/GYN. Post-graduate doctors have clinical responsibility for laboring patients, performing cesarean deliveries, and managing complications. Midwives perform normal vaginal deliveries and other nursing duties, such as medication administration and initial neonatal care. There are no additional nurses or medical assistants. Nurse-midwife staffing to patient ratios range from 1:25 during the day to 1:50 at nights and weekends [31]. There are no fees for women seeking clinical care at MRRH. A minority of women (< 1% of admissions) have the option to pay for maternity care in a private ward that is staffed separately from the main maternity unit. Current standard of care for physiologic monitoring on the maternity ward is primarily performed by manual attainment of physiologic signs by clinicians with available equipment. Based on prior audits, standard monitoring is most often performed for post-cesarean women during morning rounds by post-graduate doctors per departmental expectations. In prior work assessing frequency of monitoring, we found 2 and 4% of women had heart rate, and blood pressure respectively, checked in the first 4 h post-cesarean. Heart rate was checked in 40% of women on post op day 1, blood pressure in 38%, temperature in 7%, respiratory rate in 2% and oxygen saturation in 1% of women [31]

### Wireless physiologic monitoring system

**Fig. 1:**
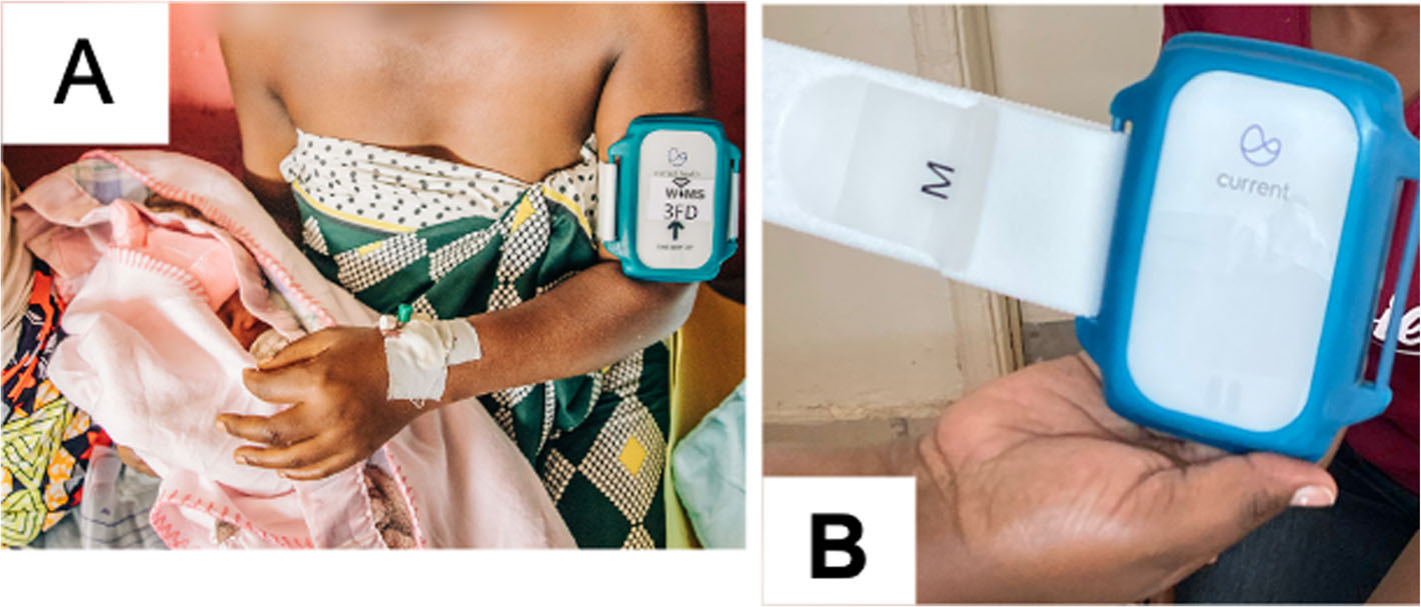
**a**: Biosensor as worn on left arm of postpartum woman. **b**: Close up of biosensor and strap

**Fig. 2:**
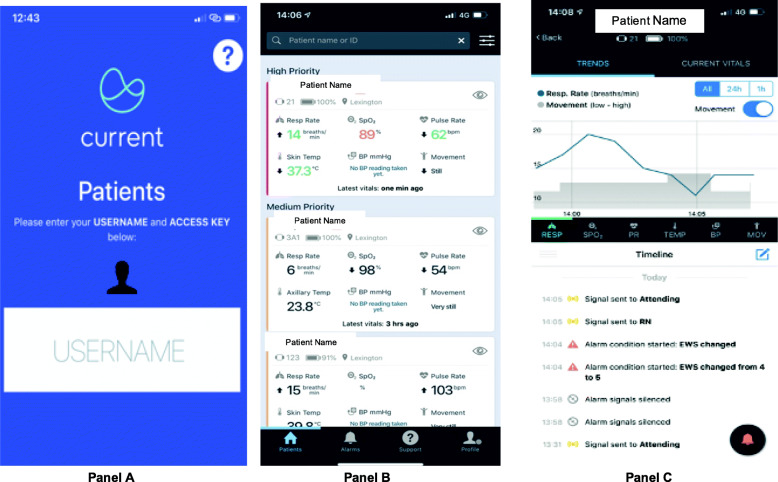
Panel **a**: Smart phone log-in interface, Panel **b**: Patient current vital signs on smart phone app; Panel **c**: Historic vital sign and timeline of previous alerts

The wireless physiologic monitoring system to be used in this study is provided by Current Health™. The biosensor used in this monitoring system has approval from the United States Food and Drug Administration for monitoring heart rate, temperature, oxygen saturation, respiratory rate and movement in adults. It has been used in a trial assessing the detection of clinical deterioration in emergency department patients [36]. The wireless monitor consists of a biosensor worn on the upper arm, preferably left arm (Fig. 1 Panel a and b). Physiologic data including heart rate, respiratory rate, temperature and oxygen saturation are captured by the monitor and transmitted via Wi-Fi to a secure cloud storage. The biosensor contains photoplethysmographic, temperature and accelerometer sensors. Measures of heart rate, respiratory rate, oxygen saturation, skin temperature and motion are derived from observations by these three sensors. Raw waveforms are transmitted from the wearable to the Current Health cloud, where they are analyzed. Blood pressure is not obtained directly from the biosensor. This physiologic sign is obtained with a separate blood pressure cuff (iHealth™). Readings from blood pressure measurements are integrated with output from the biosensor using a QR sensor on the blood pressure device and a smartphone enabled with the Current Health™ app. Once obtained, the blood pressure reading is also sent to the same cloud storage. From cloud storage, physiologic data is transmitted for the end-user access to either a web-based platform or a smart phone application (Fig. 2: Panel a). Access to either requires a username and unique pin for each user. The smart phone application is available for both Android and IOS platforms. On the web-based platform and smart phone application, physiologic data is accessible both for real-time data and historic data (Fig. 2: Panels b and c, respectively). The smartphone application allows for customizable predefined alert levels for each physiologic signs (e.g., respiratory rate > 30 breaths per minute for 5 min) or for a combination of alerts. If this parameter is met, an alert is triggered as a phone notification and requires a response from a user receiving the alert for the alert to be silenced. Users viewing historic data can also see any previous alerts that had been sent and to whom they were sent (Fig. 2: Panel c).

### Assignment of the intervention

Pregnant women will be enrolled in alternative two-week intervention and control time periods. During an intervention time period all women meeting eligibility criteria and providing informed consent will be enrolled consecutively unless biosensors are not available. With 20 biosensors available for the study and mean number of emergency cesarean deliveries of ~ 10/day, we anticipate we will be able to enroll all women meeting eligibility criteria. This allocation strategy will be used to minimize bias with sicker women being prioritized for monitoring. Randomization will not be done at the individual patient level because of a high potential for contamination (e.g., unplanned use of the monitors in control participants) and ethical concerns (e.g., control participants seeing intervention participants with potentially more clinical attention). Randomization of the intervention time periods by time will not be performed to prevent clustering of intervention time periods during certain seasons, which are particularly relevant in this rural, agrarian setting. Due to the nature of this intervention, blinding of patients, clinicians and investigators will not be feasible.

#### Intervention periods

**Fig. 3:**
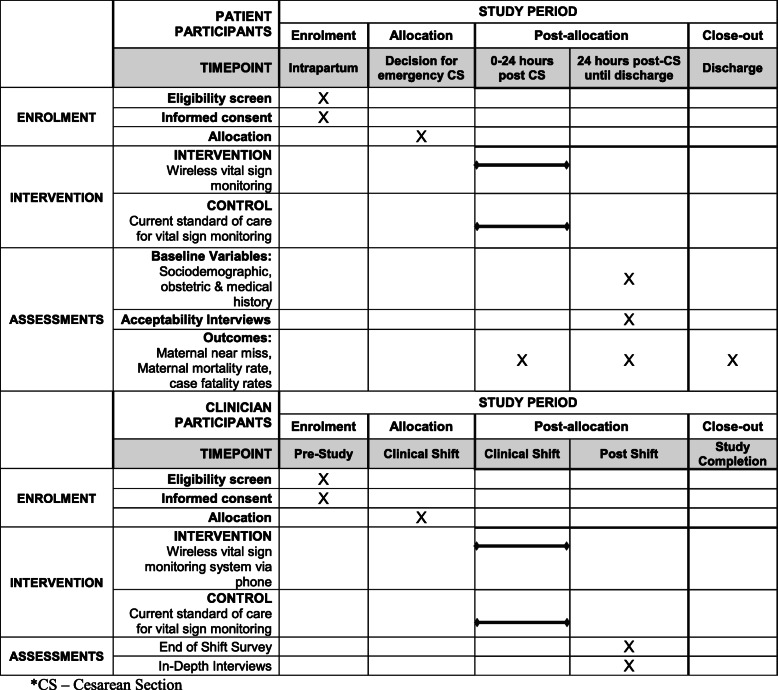
Spirit Flow Diagram

The intervention for this trial will be the use of the above-noted wireless physiologic monitoring system to monitor women for the first 24 h after completion of an emergency cesarean delivery and provide alerts to covering clinicians via a smart phone application. Eligible women consenting for the study will have the wireless monitor placed on them immediately after the completion of the cesarean delivery and then removed at the end of 24 h (Fig. 3). The monitoring system will be programmed to send alarms to phones held by clinicians with clinical coverage duties should abnormalities occur in physiologic signs. We chose limits for abnormalities in physiologic signs based on standard clinical guidelines around the common obstetric complications including hemorrhage sepsis and hypertensive disease (Table 1) [37–39].

**Table 1:** Predefined limits for clinician alerts

Physiologic sign	Cut off for alert
Heart rate (beats per minute), noted consistently for > 10 min	> 120
Systolic blood pressure (mmHg), any	> 160 or < 70
Diastolic blood pressure (mmHg), any	> 110 or < 30
Temperature (C), noted consistently for > 10 min	>38C
Respiratory rate (breaths per min), noted consistently for > 10 min	> 30

For each clinical care shift two midwives and one consultant OB/GYN will be identified and designated as the responding clinicians to receive alerts on patients. The two midwives are physically present at all times on the wards; one midwife will have responsibility for receiving alarms for covering patients on the postpartum ward and one midwife will have responsibility for coverage of alarms from patients in the operating theatres. The midwives will be provided with an emergency responder phone (Samsung J3, Android) enabled with the Current Health™ application on which they can visualize vital signs and receive notifications of abnormal physiologic signs. Consultant OB/GYNs designated as responding clinician may or may not be physically present on the ward as per current departmental policies. They will be provided with the smartphone application for download to their own personal phones, per their preference. Clinical response to alerts and notification of other clinicians (i.e., other doctors and other midwifes) will be left to the discretion of the clinician receiving the alert.

### Control time periods

The control for this study will be current standard of care for physiologic monitoring. This standard relies primarily on manual attainment of physiologic signs by clinicians with available equipment. During control periods women will not be approached or interacted with, however chart review will be performed to assess for the occurrence of the primary and secondary outcomes and to review frequency of physiologic monitoring as documented in the chart (Fig. 3).

### Eligibility criteria

#### Patient participants

Women aged 18 and older undergoing emergency cesarean delivery at MRRH are eligible for inclusion into the trial. We have restricted the target population to women undergoing emergency cesareans because of higher rates of morbidity and mortality compared to vaginal delivery and elective cesareans deliveries [40–44]. We will exclude women unable to speak Runyankole or English (two most common languages) due to study resource constraints. We will also exclude women directly admitted to the intensive care unit and the private ward due to different staffing and monitoring systems in place on those closed units.

#### Clinician participants

Midwives and consultant OB/GYNs with privileges to work on the maternity unit and with clinical duties during the study period will be eligible for participation. Postgraduate trainees in OB/GYN will not be included due to concerns for coercion with enrollment raised by the local ethics committee. Clinicians using the monitoring system and a subset of women participating in monitoring will also be eligible to participate in-depth interviews.

### Outcome Measures

#### Primary outcome

The primary outcome measure for effectiveness will be the rate of severe maternal outcome i.e. one or more of the following outcomes up until discharge. This composite measure is derived from World Health Organization (WHO) near miss morbidity criteria (Table 2) and also includes hysterectomy, cardiac arrest, prolonged unconsciousness, stroke, dialysis, intensive care unit admission and death. The WHO near miss morbidity criteria have been adopted since 2008 as a standardized approach to measure pregnancy related life-threatening conditions and are a useful tool to assess the quality of obstetric care [45, 46]. Though use in sub-Saharan Africa is limited, this measurement tool has been applied with some modification in similar settings to the proposed trial site [43, 44, 47, 48]. We chose near miss events for inclusion in the severe maternal outcome composite measure based on the ability to measure these events in both study groups (intervention and control), and as measures that should be reduced with earlier recognition of complications and subsequent intervention. Near miss events that are reliant solely on physiologic monitoring for identification will be excluded from our composite outcome as these cannot not be measured reliably in the control group.

**Table 2:** WHO Near Miss Criteria with demonstrated feasibility of collection in a RLS [28, 29, 7]

Outcome	Definition/Measurement	Included in Primary Outcome
Death	Mortality occurring at any time point after delivery and prior to discharge	X
*Clinical based near-miss criteria*		
Acute cyanosis	Blue or purple coloration of the skin or mucous membranes due to low oxygen saturation	X
Gasping	Terminal respiratory pattern, the breath is convulsively and audibly caught.	X
Severe bradypnea or tachypnea*	Respiratory rate < 6 or Respiratory rate > 40	
Shock*	Persistent systolic BP ≤ 80 mmHg or a persistent systolic BP ≤ 90 mmHg with a HR ≥ 120	
Oliguria	Urinary output < 30 ml/hour for 4 h or < 400 ml/24 h non-responsive to fluids/diuretics	X
Failure to form clots	Bedside clotting test or absence of clotting from the IV site after 7 min	X
Prolonged unconsciousness	Complete or near-complete lack of responsiveness to external stimuli	X
Cardiac arrest	Sudden absence of pulse and loss of consciousness	X
Stroke	Neurological deficit of cerebrovascular cause persisting ≥24 h	X
Uncontrollable fits	Refractory, persistent convulsions or status epilepticus	X
Total paralysis	Complete or partial paralysis of both sides of the body	X
Jaundice in the presence of pre-eclampsia	BP > 140/90 with proteinuria (> 1 + dipstick in ≥2 samples) and jaundice	X
*Laboratory-based near miss criteria*		
Acute severe azotemia	Creatinine > 300 mmol/l or > 3,5 mg/dl	X
Severe acute hyperbilirubinemia	Bilirubin > 100 μmol/l or > 6.0 mg/dl.	X
Severe acute thrombocytopenia	< 50,000 platelets/ml	X
Management-based near-miss criteria		
Use of continuous vasoactive drugs	Uninterrupted infusion of dopamine, epinephrine, or norepinephrine	X
Hysterectomy	Surgical removal of the uterus following infection or hemorrhage	X
Massive transfusion	Transfusion of ≥5 units of blood	X
Intubation and ventilation not related to anesthesia	Placement of an endotracheal tube or ventilation for > 60 min purposes other than anesthesia	X
Dialysis for acute renal failure**		X
Cardiopulmonary resuscitation	Emergency procedures including chest compressions and lung ventilation	X

#### Secondary outcome measures

Secondary outcome measures to be evaluated include the maternal mortality rate, maternal near miss rate, and case fatality rates for postpartum hemorrhage, hypertensive disease and sepsis – the three most common causes of maternal death.

To measure the number of severe maternal outcomes and other secondary outcome measures, a trained research assistant (RA) will screen the medical records of all women delivered by cesarean on a daily basis until discharge (Fig. 3). The occurrence of events will be abstracted and recorded. Chart abstraction has been used successfully to document and measure maternal near-miss criteria in settings similar to the proposed study site [47]. To minimize missing information due to missing medical records or incomplete documentation, the RA will also cross check for potential severe maternal outcomes in the defined population by screening a daily report produced by covering doctors and presented daily to the department as part of routine clinical care.

### Intervention implementation measures

The RE-AIM (*Reach, Efficacy, Adoption, Implementation and Maintenance)* framework evaluates the implementation of an intervention as a function of five factors incorporating both individual and organizational level measures (Table 3) [49–51]. This framework will be used to measure implementation.

**Table 3:** RE-AIM Implementation Science Framework [49, 50]

Metric	Dimension	Study Measure	Data source
Reach	Proportion of target population that participated in the intervention	• Percent of women with successful placement of the biosensor after delivery • Total length of time for monitoring during the 24 h after cesarean delivery • Percent of women participating in monitoring for 24 h	• Physiologic data from biosensor • Time of delivery from chart records and operating theater log books • Physiologic data recorded in charts
Efficacy	Success rate if implemented as in guidelines	• Percent of women with HR, BP, RR and Tp available at least every 4 h for 24 h after delivery • Percent of appropriate alerts sent	• Physiologic data from biosensor • End of shift survey to clinicians • Back-end data from Current Health™ • Physiologic data recorded in charts
Adoption	Proportion of practitioners adopting this intervention	• Percent of eligible clinicians participating in wireless monitoring • Number of clinical actions in response to alerts	• End of shift survey to clinicians • Back-end data from Current Health™
Implementation	Extent to which the intervention is implemented in the real world	• Fidelity of implementation to the study protocol	• Documentation of any necessary adjustment to the protocol after study enrollment begins • Documentation of disruptions due to external factors
Maintenance	Sustainability of program over time	• Sustainability of reach, efficacy and adoption as measured by stability of measures over study period	• Above sources reviewed over the 12-month study period

*HR -Heart Rate, BP – Blood Pressure, RR – Respiratory Rate, Tp – Temperature

During enrollment of women into wireless physiologic monitoring, an RA will monitor and record process measures around the use of the wireless monitoring system including: 1) proportion of women with placement of the biosensor after cesarean delivery (*Reach),* 2) total length of time for monitoring over 24 h (*Reach)*. All patient participants in both intervention and control time periods will have chart abstraction by RAs to document frequency of physiologic sign documentation (*Reach)*. At the end of each shift, clinicians are sent a brief survey to document if they logged into the system (*Adoption),* received an alert (*Efficacy)*, the time they became aware of the alert (*Efficacy)* and any clinical action taken as a result (*Adoption)*. Back end data from the Current Health™ application will also be reviewed to document, logins to the system, receipt of automated alerts, time of receipt and type of alert received *(Efficacy, Adoption, Implementation)* and acknowledgement of the alert *(Adoption)*. RAs will also document on a daily basis external factors such as electricity outage, wireless disconnections, strikes, clinical supply stock outs that may impact study procedures and clinical use of the system *(Implementation)*.

To provide context to the above quantitative measures and to understand acceptability and facilitators and barriers to uptake of wireless physiologic monitoring, semi-structured interviews will be performed with clinicians using the monitoring system and postpartum women undergoing monitoring. Up to 35 clinicians will be recruited for semi-structured interviews. Based on current staffing at MRRH, this will likely include all clinicians that enroll in the study. Up to 30 postpartum women who wear the biosensor will be recruited for interview, purposively sampled for age (18–34 and > 35 years) and level of use of the biosensor (~ 15 women who completed 24 h of monitoring and ~ 15 women with less than 24 h of monitoring). If the thematic saturation is not reached within each stratum with this sampling, additional women will be interviewed resources permitting. Interviews will be conducted 6 months after enrollment begins. This will allow for 6 intervention time periods to be completed and reduce potential for the learning curve associated with a new system to influence clinician perspectives. Interviews will be conducted until thematic saturation is reached. The interview guides for clinicians and postpartum women will be developed in a multi-step fashion. The initial interview guide will be developed through a review of existing literature, including the Technology Acceptance Model [52–56] as a framework and adaptations to this model for use in resource-limited settings [53], as well as input from local OB/GYNs and other study team members. This interview guide will be piloted on 3 doctors and five women. The initial guide will then be revised as needed based on the pilot phase. Initial interview guides are available in Supplemental file 1. An RA trained in qualitative interview technique and fluent in the language of choice of the participants (either Runyankole or English) will administer interviews, perform verbatim transcriptions, and translate into English where necessary. Interviews will be conducted in a private space and designed to last less than one hour. For postpartum women, interviews will be performed on postpartum day 2 or 3, at a time convenient to the woman.

### Sample size

On average, approximately 8–10 emergency cesarean deliveries are performed at MRRH daily. Thus, in a two-week time period we estimate ~ 112 emergency cesareans performed. We plan an intention to treat approach and plan to capture outcomes on all eligible women delivered by emergency cesarean whether or not they have monitoring as per protocol. This yields an estimated 112 women per time period. Over 12 months of enrollment, we will therefore have 13 intervention time periods and 13 control time periods This yields an estimated 1456 women in the intervention group and 1456 in the control group. Assuming an intraclass correlation coefficient of 0.01, the effective sample size after taking account of clustering is 633 per group. This sample size will allow us to detect a difference of 5.7% in the severe maternal outcome rate between the two study arms, with a two-sided significance level of 0.05, and 80% power, assuming a baseline rate of severe maternal outcome of 13% [43, 47].

### Data management

All data captured will be entered into REDCap (Research Electronic Data Capture), which is a secure, web-based software platform designed to support data capture for research studies [57, 58]. In depth interviews will be digitally recorded and then transcribed and translated within 72 h of the interview. Transcripts will be stored in a secure file sharing system. Identifiable information will only be used for study logistics and management and accessible to the core study investigators and research staff. Data for analysis will be de-identified.

### Analysis

#### Intervention effectiveness

We plan an intention to treat approach to assess effectiveness. We will calculate the event rates for both primary and secondary outcomes during intervention and control time periods on all eligible women during those time periods. These will be compared using Poisson regression models. While intervention allocation was at the level of the two-week time period, the analysis will be at the individual patient level adjusting for clustering of observations within time periods.

Exploratory analyses will be used to determine if the intervention has a more significant effect in certain subgroups of women (e.g. women < 35, or women with more education). The interaction between study arm and comorbidities (i.e., HIV, hypertension, pulmonary, cardiac or kidney disease, malaria) will also be tested in the logistic regression models. Subgroup effects will be reported if there is strong evidence of heterogeneity of intervention effect.

#### Intervention implementation

Implementation will be assessed using the RE-AIM framework. Data will be assessed at 4-month intervals (i.e. after 4 intervention time periods) and used to inform changes in alert notifications to optimize clinical adoption. Data from the entire study period will be used to assess implementation outcomes. For example, if frequent false alarms are noted at the above thresholds these will be adjusted if needed. In depth interviews will be analyzed using a grounded theory approach. Specifically, data will be analyzed using content analysis, in an iterative, multi-step process [59, 60]. Transcripts will be reviewed for key concepts and used to develop a codebook. Approximately 20% of transcripts will be double coded to ensure consistency with the codebook. Coded data will be used to develop descriptive categories. While we will compare these descriptive categories to the technology acceptance model as a framework for interpretation, we will also be interested to explore new themes that may emerge and provide context to our understanding of clinical adoption as measured by the RE-AIM framework.

### Human subjects protection

#### Informed consent

Women undergoing emergency cesarean delivery during intervention time periods will be approached for written consent for them to wear the biosensor for 24 h after delivery. We will approach eligible pregnant women for informed consent prior to clinically indicated cesarean delivery but after the clinical decision is made and clinical consent for the cesarean is obtained (Fig. 3). Based on clinical experience at this site, the average time from the clinical decision to initiation of the cesarean delivery is often greater than one hour, thus allowing time for research informed consent to be obtained [61]. For women who do not have the capacity to consent due to clinical status, a health care proxy will be approached. Written informed consent will be performed prior the cesarean delivery to allow monitoring to begin immediately after the procedure. A trained research nurse will obtain informed consent. Consent is waived for chart review of women undergoing emergency cesarean delivery during control periods.

Clinicians eligible for enrollment will learn about the study and eligibility for participating during routine staff meetings. Study investigators will provide further details and explanation of the study procedures to clinicians interested in participating and obtain written informed consent for clinicians willing to participate.

#### Ethics and research governance

The trial has institutional regulatory board approval from the Partners Healthcare Institutional Regulatory Board, Boston, MA (2019P000885), Mbarara University of Science and Technology Research Ethics Committee (17/10–18) and the Uganda Council of Science and Technology (HS417ES). Written consent is obtained from both women undergoing wireless monitoring and clinician participants. Any amendments to the trial will be communicated and approved by all three ethics boards. A data and safety monitoring board will oversee the study. The board will meet twice during the study (month 4 and 8 of enrollment) and once at the end. The board will review if there is a signal of higher rates of severe maternal outcome than expected to determine if these are related to the study intervention and then provide recommendations to study changes including but not limited to potential consideration of study termination. We will also ask their assistance in monitoring study implementation, including participant burden.

## Discussion

Improving quality of care during facility-based childbirth is a global health priority. Without vast increases in the number of health care workers, current standards for inpatient physiologic monitoring will be unattainable. Furthermore, current projections are that shortages in health providers are likely to worsen, rising from a shortfall of 7.3 million currently to 18 million by 2030, and with deficits concentrated in resource-limited settings within sub-Saharan Africa and South-east Asia [62]. As such, new and innovative strategies are needed to provide the necessary and recommended close monitoring for women seeking obstetric care in facilities despite staffing limitations. This study will provide insights into the effectiveness and ability to use wireless physiologic monitoring to overcome human resource limitations.

In this trial we describe the use of a hybrid effectiveness-implementation design. Hybrid designs that simultaneously combine clinical effectiveness trials with implementation evaluation blend both designs with the goal of supporting rapid translation into routine practice [63]. This study design is particularly relevant in resource-limited settings where strategies from other settings may perform differently yielding different outcomes, and yet should effectiveness be proven, rapid uptake into practice is valuable. The proposed intervention, i.e. the use of wireless technology for physiologic monitoring in resource-limited settings, has strong face validity, a strong base of indirect evidence suggesting effectiveness, and minimal risk associated with the intervention; it thus meets conditions recommended for hybrid designs [63]. Implementation outcomes will provide a richer understanding of the results from the effectiveness component of the study as well as providing the platform for scale up and adoption of this intervention into routine clinical care, if proven effective.

Findings from this trial will provide preliminary data to design a prospective cluster randomized, controlled intervention trial using wireless physiologic monitoring coupled with an mHealth alert system to reduce facility-based maternal mortality and morbidity. Additionally, findings from this trial may inform efforts to expand monitoring to other groups of women e.g. laboring women, or high-risk women (e.g. women with pre-eclampsia or eclampsia) who have a vaginal delivery.

## Trial status

Protocol version date: 05/28/2019. Protocol version: 2.

We began enrollment into the trial began on 21st January 2020 and initially projected to end 20th January 2021. Due to the COVID-19 pandemic, study activities were put on hold on March 3rd, 2020. Study activities resumed in September 2021 in line with local public health control measures and recommendations and projected to enroll until July 2021.

## Supplementary Information

**Additional file 1.**

## Data Availability

The datasets to be generated and used in the study will be available from the corresponding author on reasonable request after completion of the study and primary publications.

## Abbreviation

RE-AIM Framework: Reach, Efficacy, Adoption, Implementation, Maintenance Framework.
