# Supplementary material for: Wireless versus routine physiologic monitoring after cesarean delivery to reduce maternal morbidity and mortality in a resource-limited setting: protocol of type 2 hybrid effectiveness-implementation study
Source: BMC Pregnancy Childbirth. 2021 Feb 12;21:124. doi: 10.1186/s12884-021-03550-w (PMC7880025; doi:10.1186/s12884-021-03550-w)
Supplement: Supplementary file 1 — Additional file 1. [file 12884_2021_3550_MOESM1_ESM.pdf]

Postpartum Woman Interview Guide

Reminder to Interviewer:

Questions in the left-hand column will be asked of all participants, while questions in the right-hand columns are probes, and are to be asked only to help the participant describe her/his experience **or** if the participant does not bring up these topics spontaneously

> ***Instructions****: Hello my name is [Interviewer name]. I am research assistant with the Wireless Monitoring Study. We are conducting interviews with several women to understand how women found using the wireless monitor. I will now ask you several questions about the monitoring device you used over the last day. Please tell me what you think, not what you think we want to hear. This interview will last up to* 60 minutes. *Let me know if you would like to take a break or have any questions or concerns. I will digitally record this interview for later transcription. This information will be completely confidential and not impact your care here or at any other facility. You may stop this interview at any time. If you are bothered by a question and do not wish to answer, please let me know.*

Topics	***Probes***
**1. I'd like to ask you some questions about your general thoughts on the device. First tell me your general thoughts of the monitoring device you were asked to wear.**	*Tell me about the way it felt to wear the device.*<br>*Tell me about the way you felt about the design of the device. Be design I mean tell me about how you felt about the shape, size, weight, and look of the device.*<br>*Tell me about where you had to wear the device on your body.*<br>*Tell me about the length of time you had to wear the device.*<br>What did you like about the device?<br>What did you not like about the device?
**2. How do you think the device may have influenced your care from doctors? From nurses?**	*How did doctors/nurses explain the device to you?*<br>*In what ways did you think doctors/nurses may have treated you because you had the device?*<br>*How did you feel knowing the device was continuously monitoring you?*<br>*How did you feel knowing the doctors/nurses could tell some information about you without being there?*
**3. How do you think the device may have affected the way other women treated you?**	How did you feel wearing the device affected your privacy?
4. **I'd like to ask you questions about how the device may have affected your thoughts about your baby. First tell me *about any concerns you had about the device and the baby***	*Tell me about any physical impact the device had on how you held your baby*<br>*Tell me about any physical impact the device had on how you breastfed your baby*<br>*What could be different about the device that may make it better to hold your baby*<br>*What could be different about the device that may make it better to breastfeed?*

5. **What problems did you face in wearing the device?**	*How did you fix/solve any problems with wearing the device?*<br>*What would you change about the device to avoid these problems?*
6. **What would you tell other women undergoing childbirth and delivery about the device? Why?**	*How you describe the experience of wearing the device to other women?*
7. **Please tell me what else we should know about the monitoring device?**	
**To Interviewer: Please summarize interview and ask for feedback from the participant**<br>This is what I have heard from you [summarize findings] is there anything that is not correct or that you would like to add to?	

Clinician Interview Guide

Reminder to interviewers:

Questions in the left-hand column will be asked of all participants, while questions in the right-hand columns are probes, and are to be asked only to help the participant describe her/his experience **or** if the participant does not bring up these topics spontaneously

Instructions: *Hello my name is [Interviewer name]. I am research assistant with the Wireless Monitoring Study. We are conducting interviews with several clinicians to understand what worked and what didn't with the wireless monitoring system and how it can be improved. [I will ask you several questions about the monitoring system you have been using Please tell me what you think, not what you think we want to hear. This interview will last up to* 60 minutes. *Let me know if you would like to take a break or have any questions or concerns. I will digitally record this interview for later transcription. This information will be completely confidential and not impact your training here or at any other facility or any evaluations. You may stop this interview at any time. If you are bothered by a question and do not wish to answer, please let me know.*

**Topics and Main Questions**	**Probes**
**1. I'd like to ask you some questions about your general thoughts on the monitoring system. First tell me your general thoughts of monitoring system.**	*Tell me about how it felt to use the system*<br>*Tell me about the way you felt about the overall design of the system? By design I mean the shape, size, look of the sensor.*<br>*Tell me about the way you felt about the biosensor and placing it on patients?*<br>*How did patients respond to you placing it on them?*<br>*Tell me about the way you felt about the central monitor and inputting the patient details*
**2. How do you feel about having to carry the responder phone?**	*Tell me about how having the phone may have helped you?*<br>*Tell me about how having the phone may have been difficult?*
3. I'd like to ask you some questions about any alerts received on the phone? First tell me your general thoughts about the messages?	*Tell me about how it felt to receive a message? What emotions did you feel when you received the alert?*<br>*Tell me about the information contained in the message*<br>*Tell me about how you reacted after receiving a message*<br>*Tell me about a time when you did not act on an alert and why?*<br>*Tell me about a time when did act on alert and why*<br>How did carrying the phone and receiving alerts affect your other clinical duties?<br>*How did you feel about the accuracy of the alerts?*<br>What other things may be useful to know in an alert?
4. How do you think the device may have influenced your care of women wearing the biosensor during your call?	How did having the system change how you performed your clinical duties.
**5. What problems did you face in using the monitoring system?**	*How did you fix/solve any problems with the monitoring system?*

	What would you change about the monitoring system to avoid these problems?
**6. What did you like about the monitoring system?**	*What are the things that made it helpful?*
**7. How did you think women wearing the monitors thought about the monitoring system?**	*What do you think they liked about it?*<br>*What do you think they didn't like about it*<br>*What do you think would make it better for hem*
**8. What would you tell other doctors who were asked to use the monitoring system?**	
**9. What would you need outside of a research study to make this monitoring system work all the time?**	
10. **Please tell me what else we should know about the monitoring system?**	*What would have made the monitoring system better?*<br>*What would have made it more suitable for this environment?*
**To Interviewer: Please summarize interview and ask for feedback from the participant**<br>This is what I have heard from you [summarize findings] is there anything that is not correct or that you would like to add to?	
